# Supplementary material for: The association between tropical cyclones and dengue fever in the Pearl River Delta, China during 2013-2018: A time-stratified case-crossover study
Source: PLoS Negl Trop Dis. 2021 Sep 9;15(9):e0009776. doi: 10.1371/journal.pntd.0009776 (PMC8454958; doi:10.1371/journal.pntd.0009776)
Supplement: S1 Fig — (PDF) [file pntd.0009776.s001.pdf]

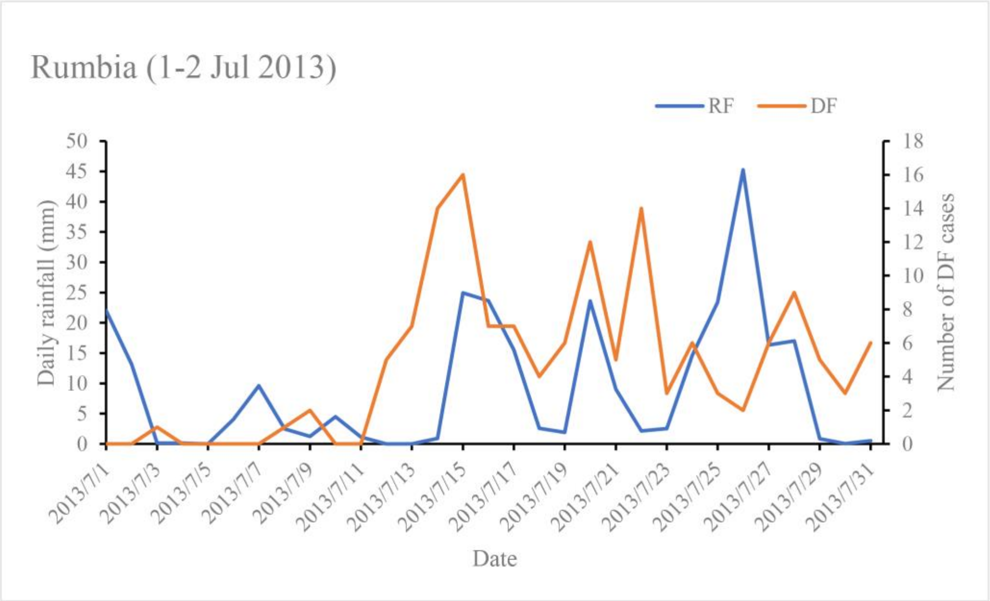
Rumbia (1-2 Jul 2013)
RF
DF
Daily rainfall (mm)
Number of DF cases
Date

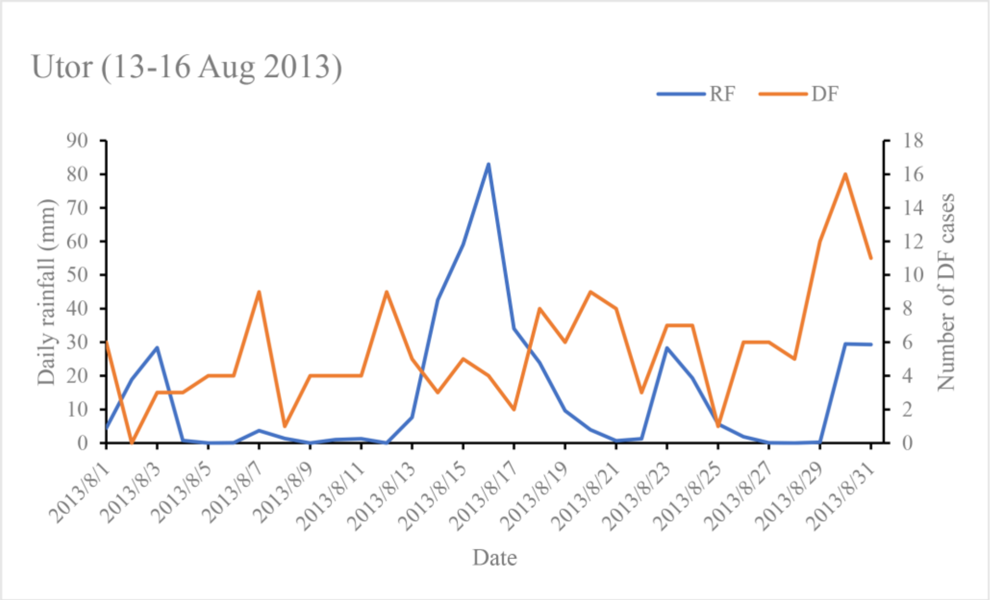
Utor (13-16 Aug 2013)
RF
DF
Daily rainfall (mm)
Number of DF cases
Date

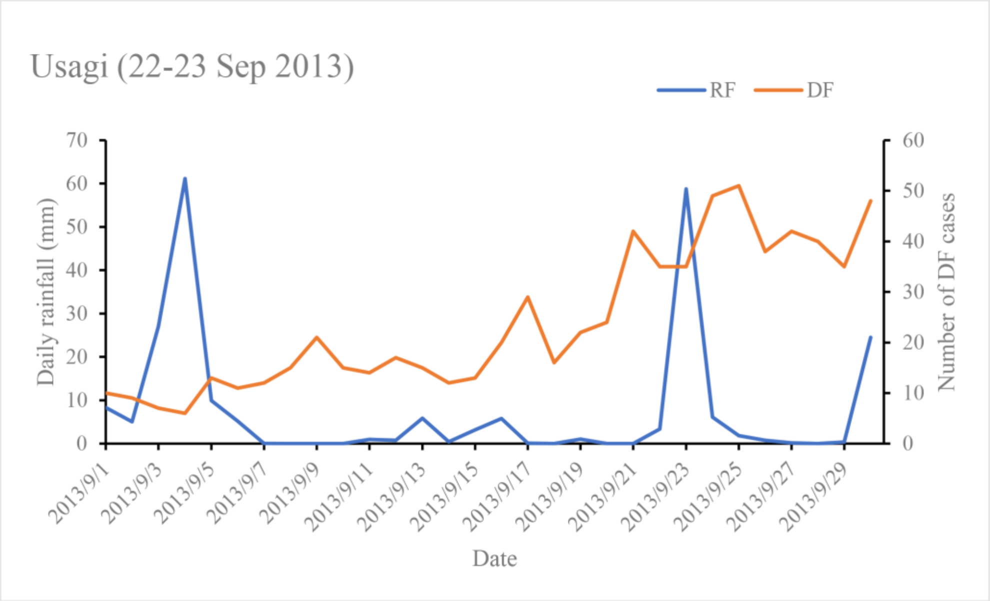
Usagi (22-23 Sep 2013)
RF
DF
Daily rainfall (mm)
Number of DF cases
Date

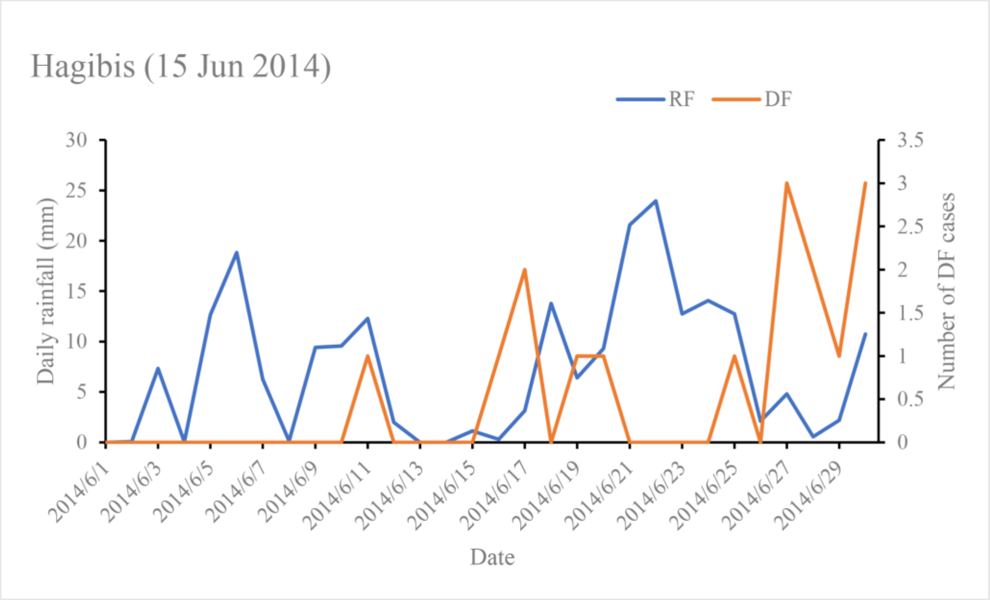
Hagibis (15 Jun 2014)
RF
DF
Daily rainfall (mm)
Number of DF cases
Date

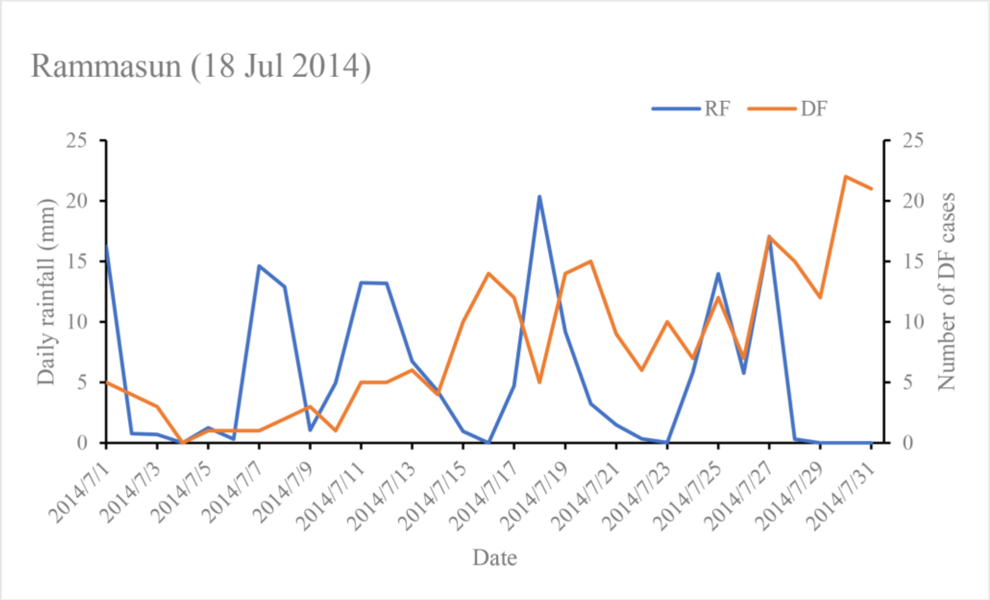
Rammasun (18 Jul 2014)
RF
DF
Daily rainfall (mm)
Number of DF cases
Date

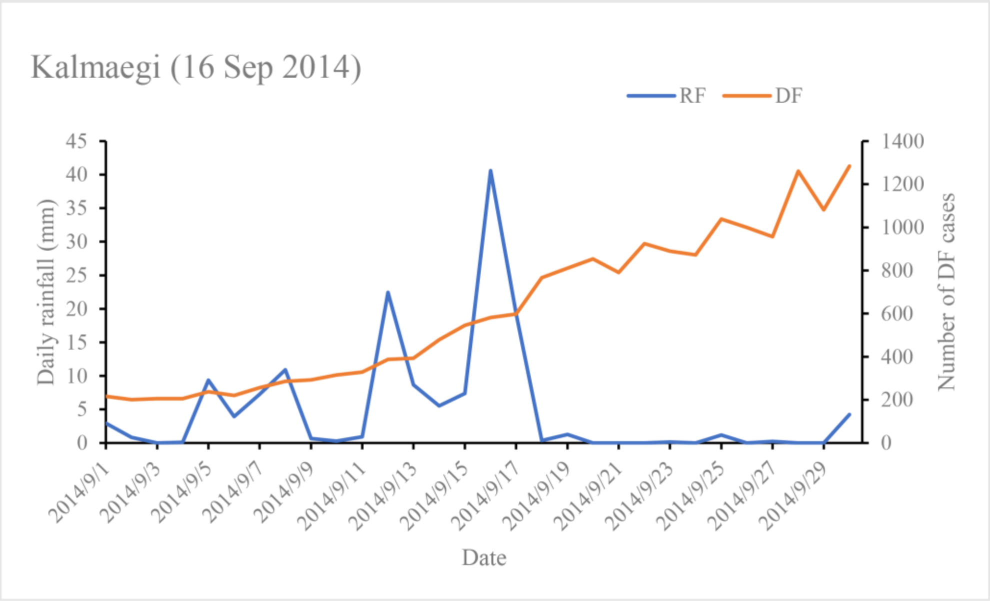
Kalmaegi (16 Sep 2014)
RF
DF
Daily rainfall (mm)
Number of DF cases
Date

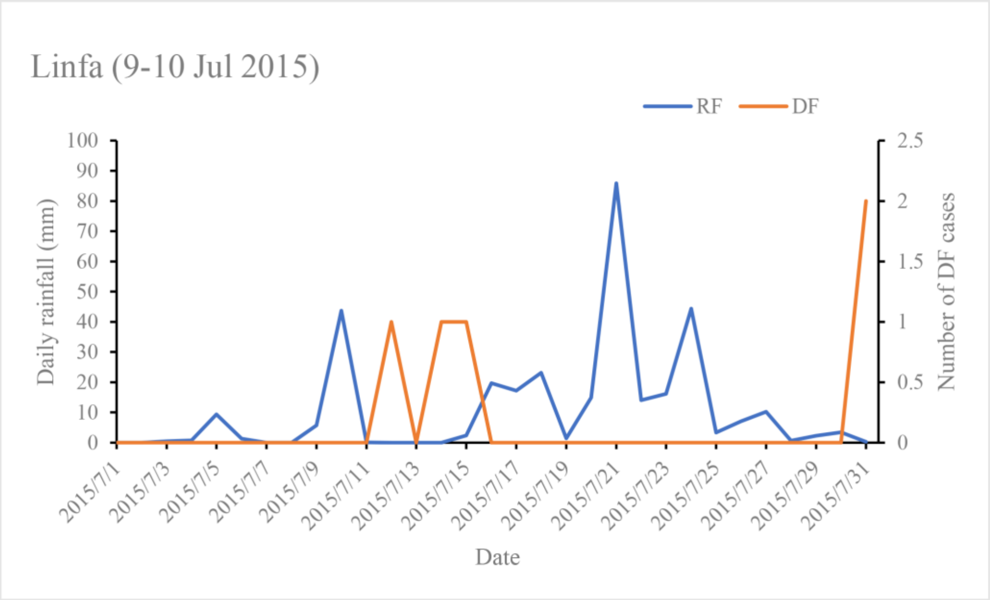
Linfa (9-10 Jul 2015)
RF
DF
Daily rainfall (mm)
Number of DF cases
Date

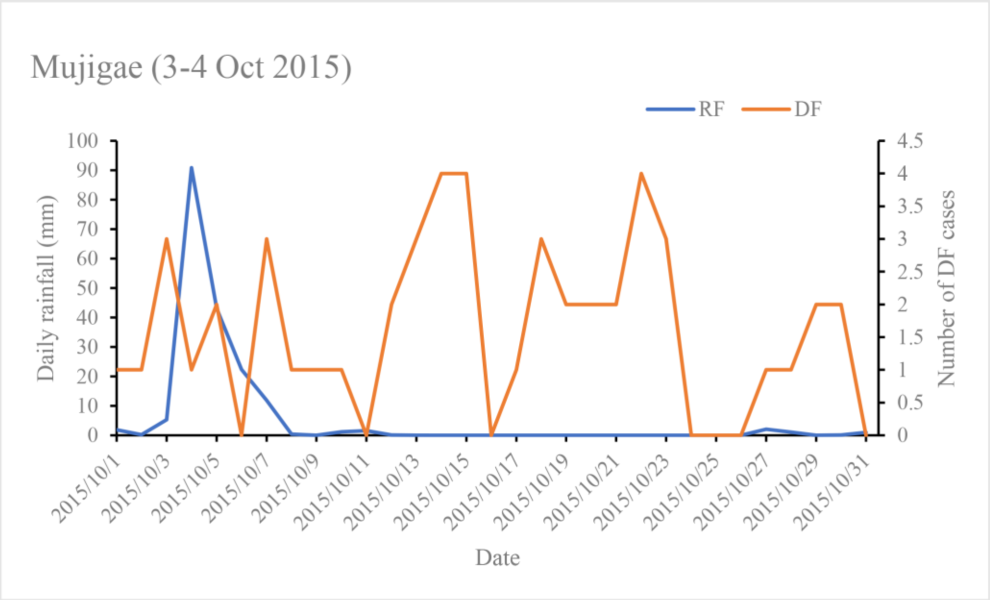
Mujigae (3-4 Oct 2015)
RF
DF
Daily rainfall (mm)
Number of DF cases
Date

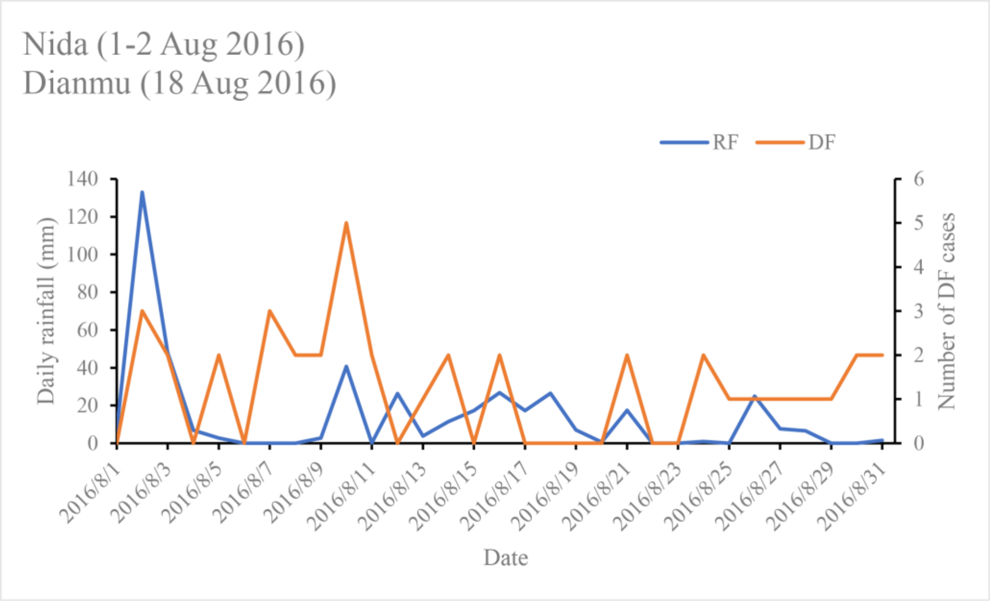
Nida (1-2 Aug 2016)
Dianmu (18 Aug 2016)
RF
DF
Daily rainfall (mm)
Number of DF cases
Date

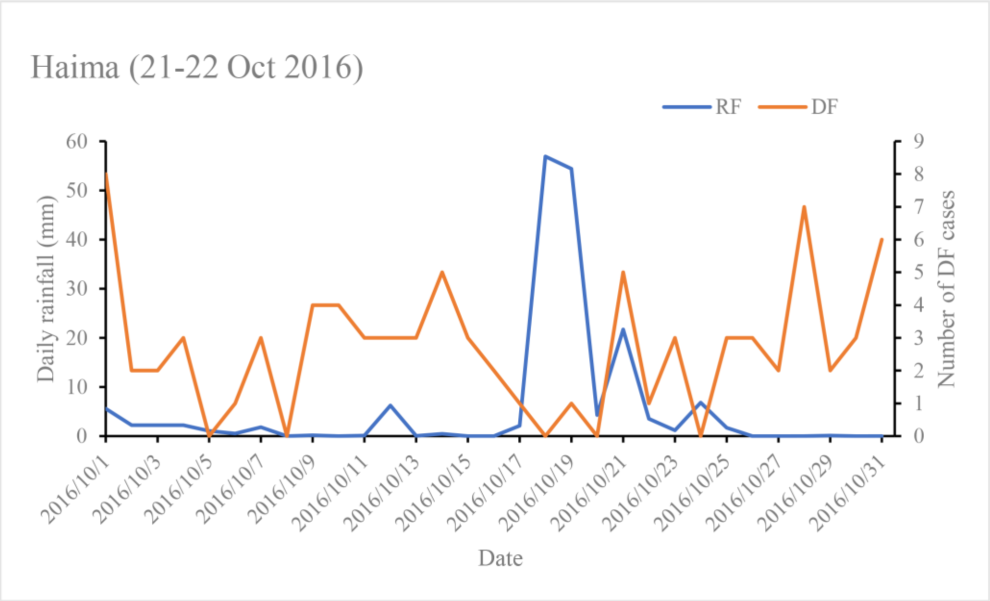
Haima (21-22 Oct 2016)
RF
DF
Daily rainfall (mm)
Number of DF cases
Date
60
50
40
30
20
10
0
9
8
7
6
5
4
3
2
1
0
2016/10/1
2016/10/3
2016/10/5
2016/10/7
2016/10/9
2016/10/11
2016/10/13
2016/10/15
2016/10/17
2016/10/19
2016/10/21
2016/10/23
2016/10/25
2016/10/27
2016/10/29
2016/10/31

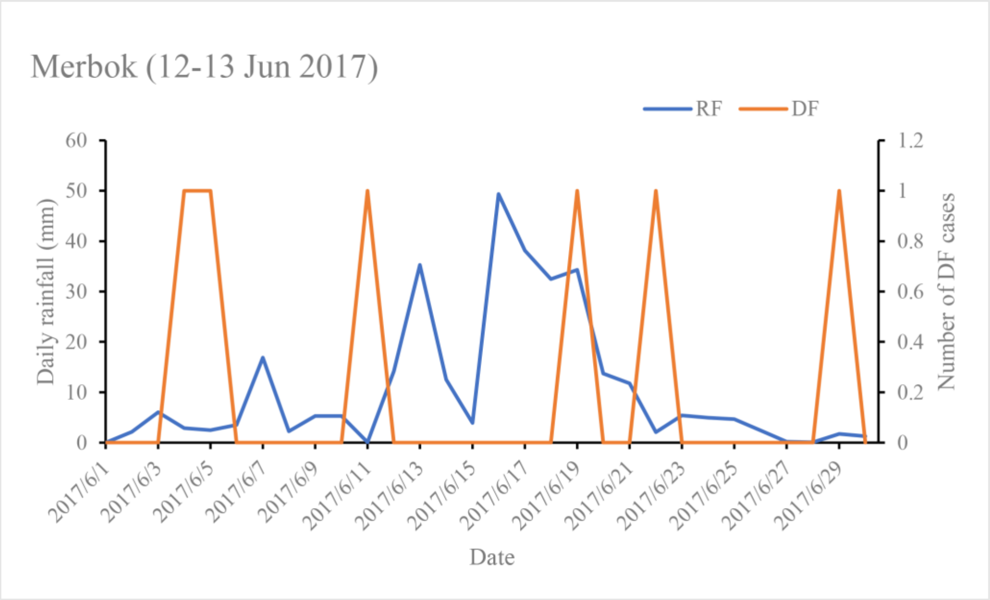
Merbok (12-13 Jun 2017)
RF
DF
Daily rainfall (mm)
Number of DF cases
Date
60
50
40
30
20
10
0
1.2
1
0.8
0.6
0.4
0.2
0
2017/6/1
2017/6/3
2017/6/5
2017/6/7
2017/6/9
2017/6/11
2017/6/13
2017/6/15
2017/6/17
2017/6/19
2017/6/21
2017/6/23
2017/6/25
2017/6/27
2017/6/29

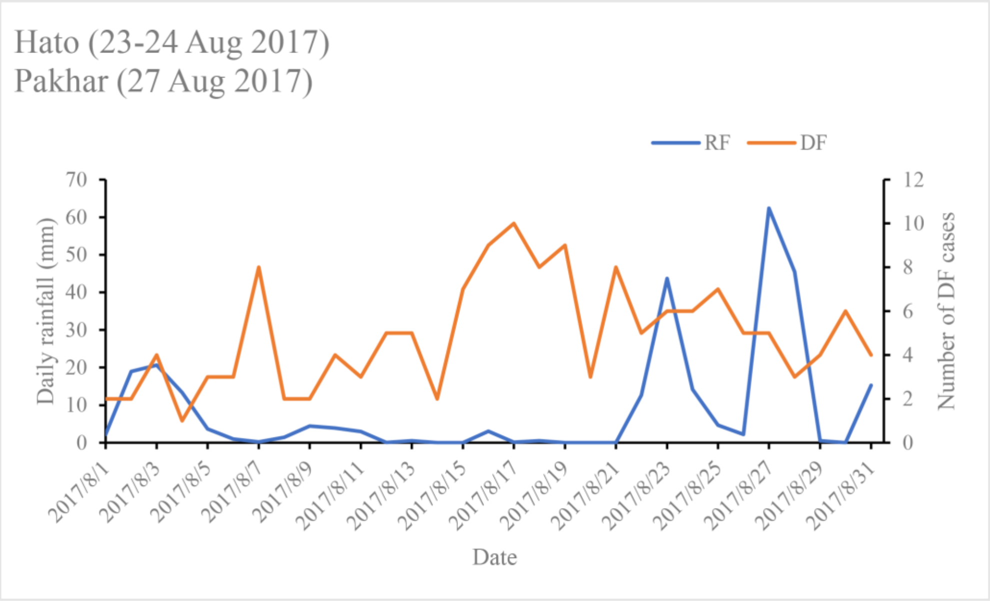
Hato (23-24 Aug 2017)
Pakhar (27 Aug 2017)
RF
DF
Daily rainfall (mm)
Number of DF cases
Date
70
60
50
40
30
20
10
0
12
10
8
6
4
2
0
2017/8/1
2017/8/3
2017/8/5
2017/8/7
2017/8/9
2017/8/11
2017/8/13
2017/8/15
2017/8/17
2017/8/19
2017/8/21
2017/8/23
2017/8/25
2017/8/27
2017/8/29
2017/8/31

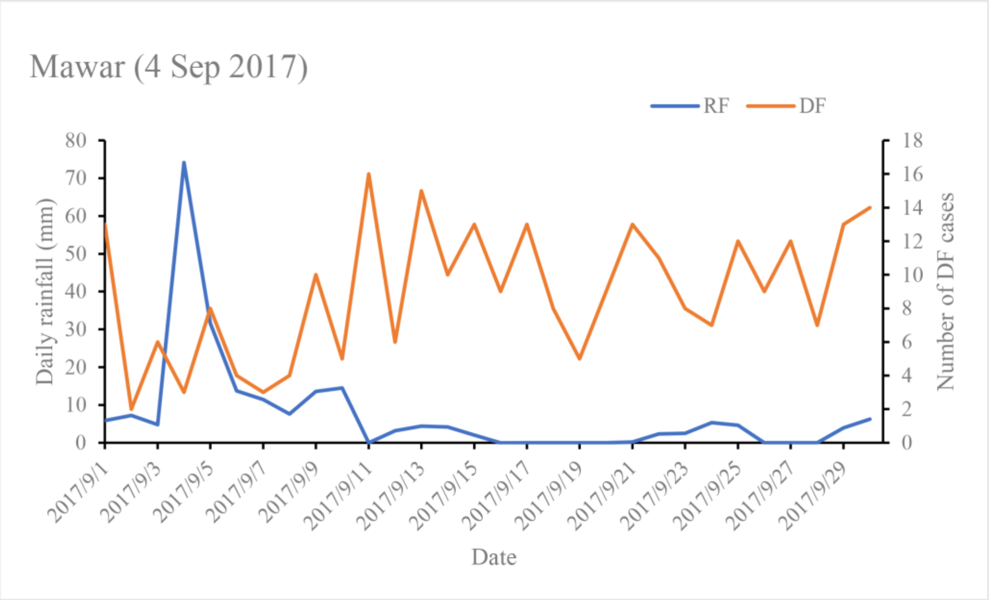
Mawar (4 Sep 2017)
RF
DF
Daily rainfall (mm)
Number of DF cases
Date

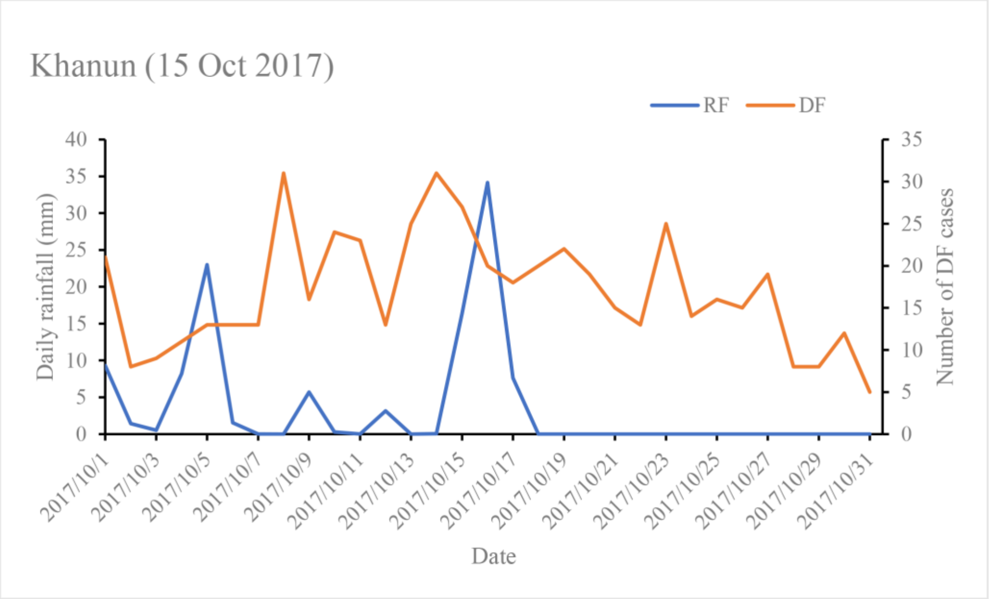
Khanun (15 Oct 2017)
RF
DF
Daily rainfall (mm)
Number of DF cases
Date

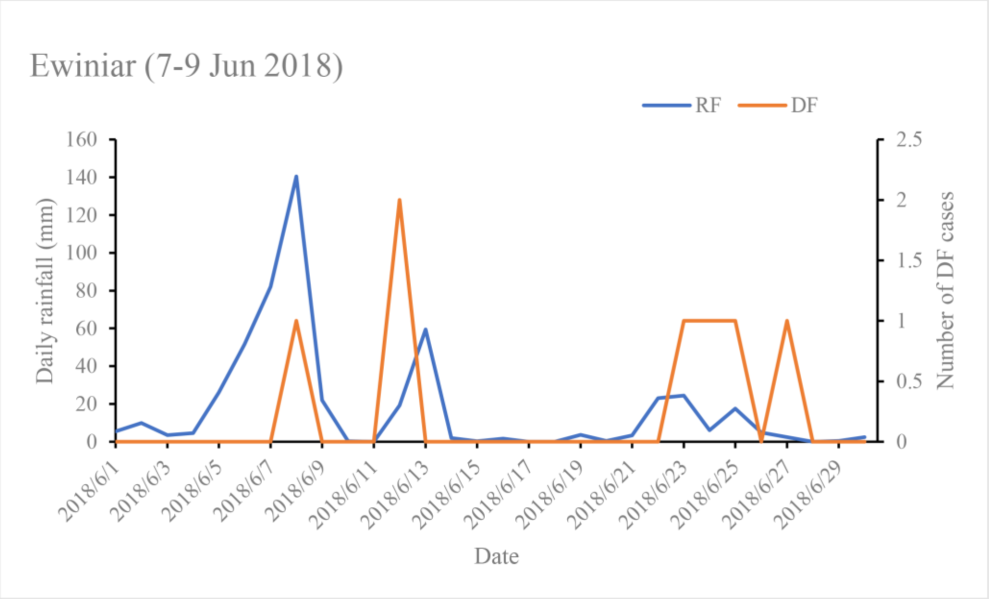
Ewiniar (7-9 Jun 2018)
RF
DF
Daily rainfall (mm)
Number of DF cases
Date

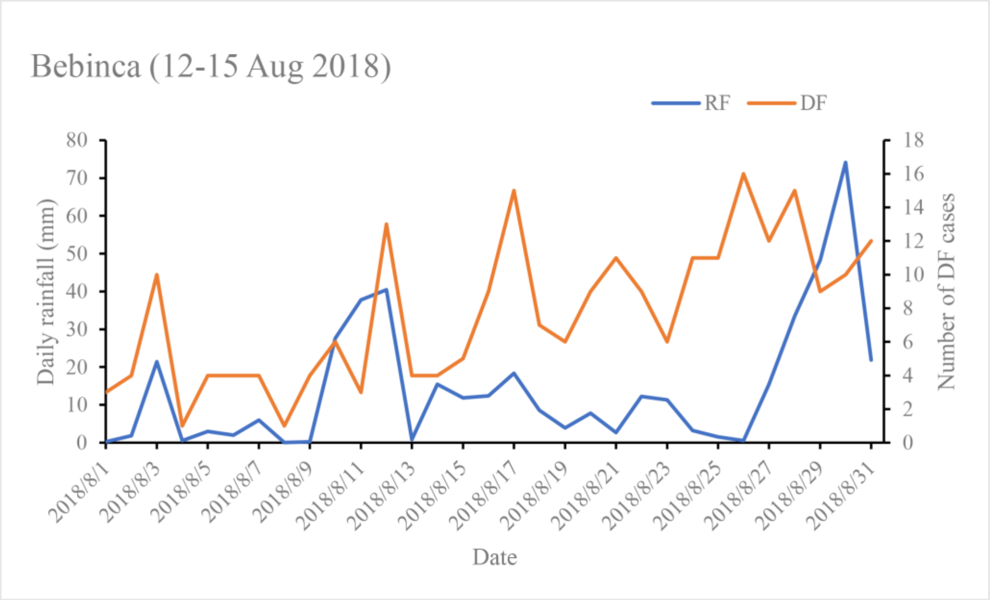

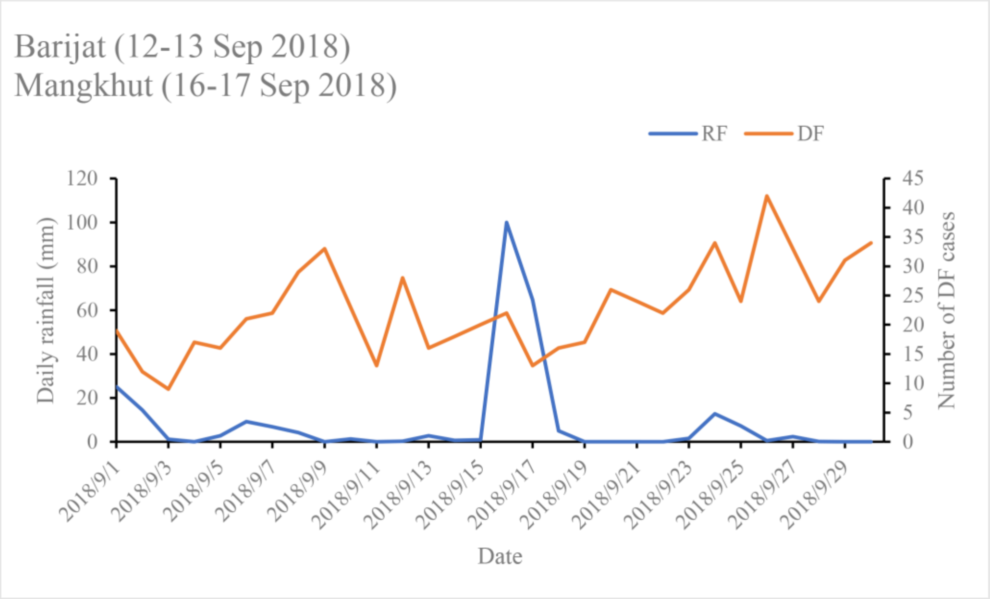

S1 Fig. Daily rainfall and number of DF cases in study periods of each tropical cyclone in the PRD from 2013 to 2018.
