# Supplementary figures and images for: The association between tropical cyclones and dengue fever in the Pearl River Delta, China during 2013-2018: A time-stratified case-crossover study
Source: PLoS Negl Trop Dis. 2021 Sep 9;15(9):e0009776. doi: 10.1371/journal.pntd.0009776 (PMC8454958; doi:10.1371/journal.pntd.0009776)

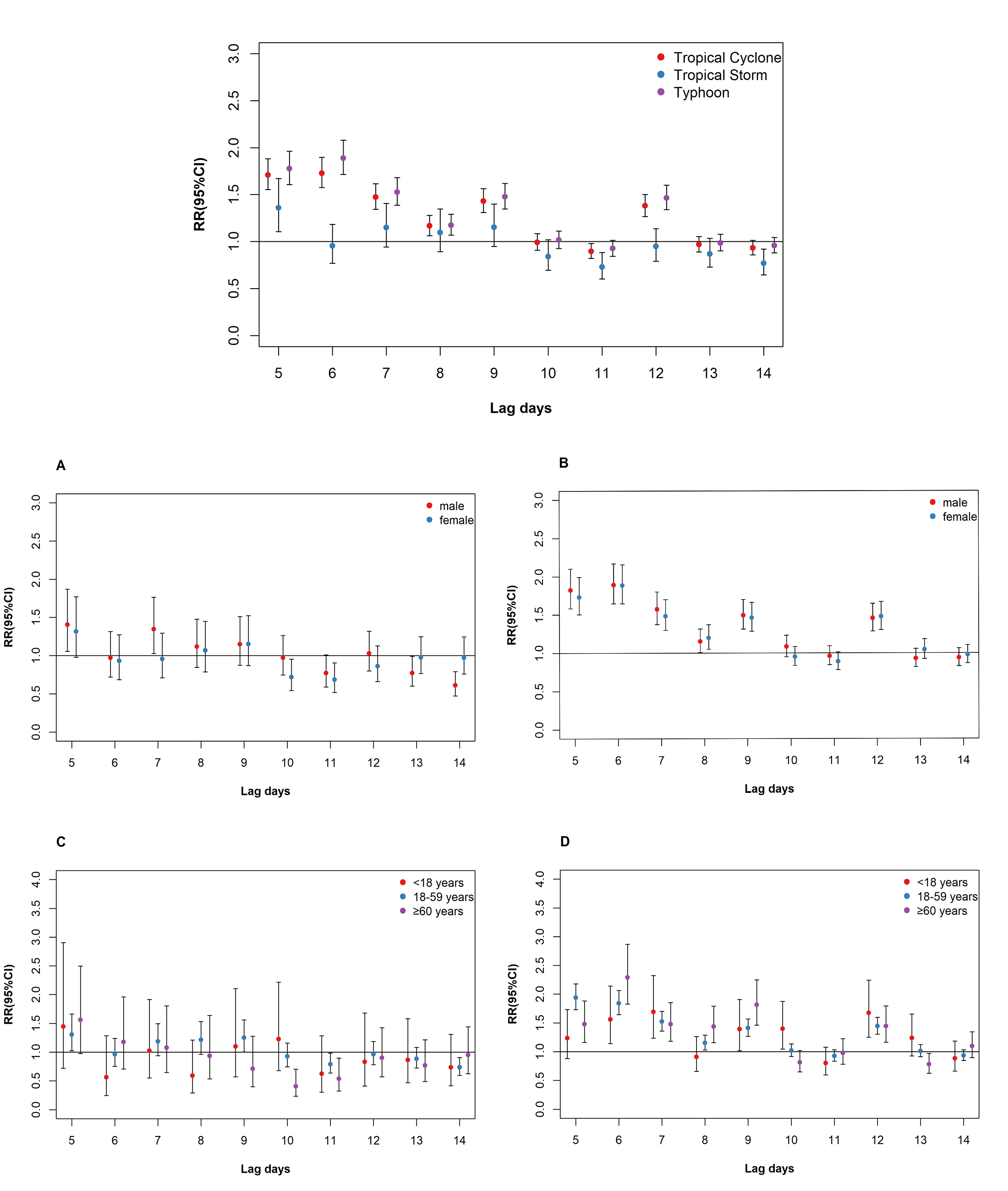

Supplement: S2 Fig — (TIF) [file pntd.0009776.s002.tif]

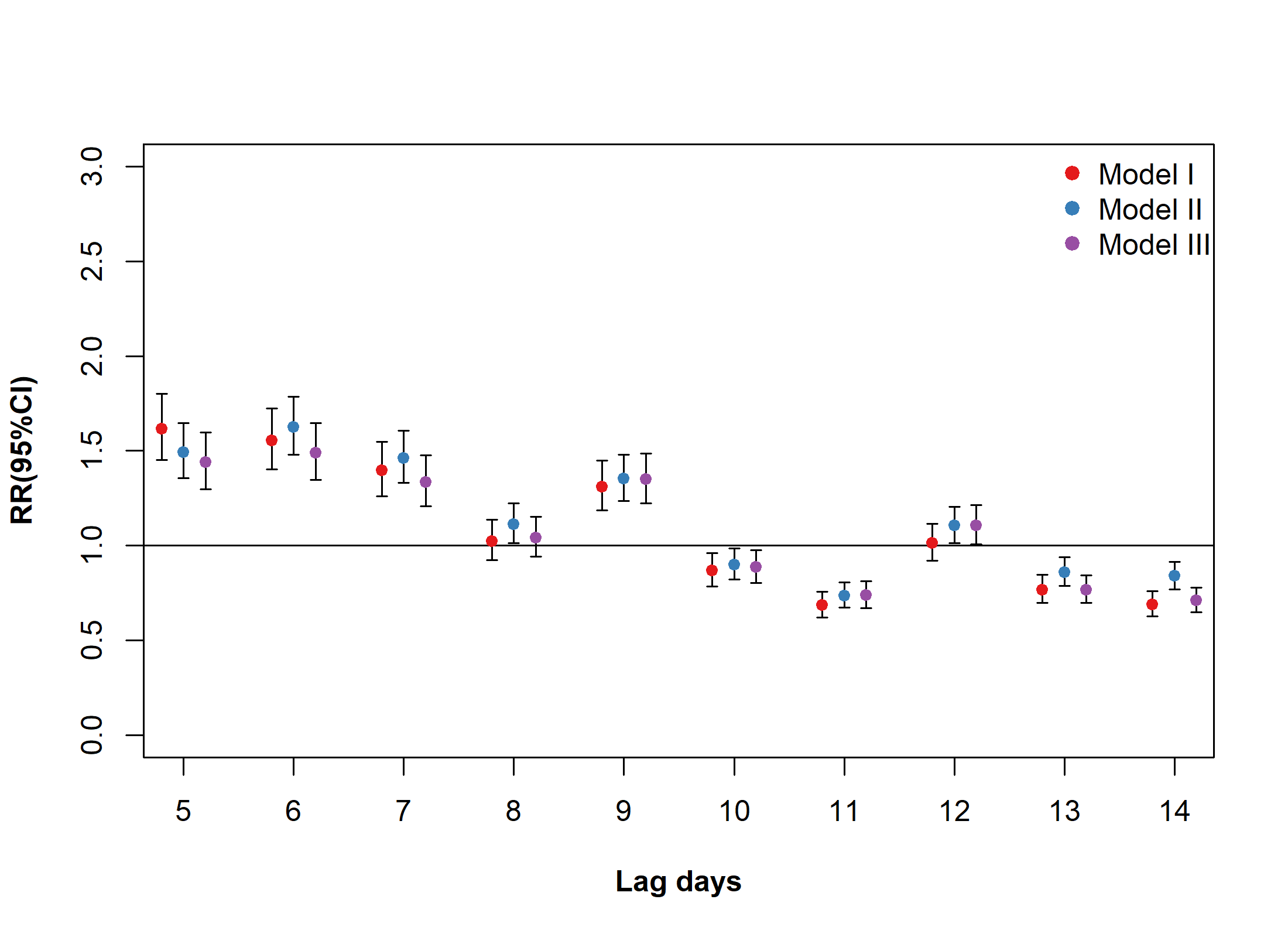

Supplement: S3 Fig — Model I: original model; Model II: excluding RF; Model III: excluding EWV. (TIF) [file pntd.0009776.s003.tif]
