# Supplementary material for: The association between tropical cyclones and dengue fever in the Pearl River Delta, China during 2013-2018: A time-stratified case-crossover study
Source: PLoS Negl Trop Dis. 2021 Sep 9;15(9):e0009776. doi: 10.1371/journal.pntd.0009776 (PMC8454958; doi:10.1371/journal.pntd.0009776)
Supplement: Attachment — Submitted filename: Response to Reviewers.docx [file pntd.0009776.s006.docx]

**Response to Reviewers’ Comments**

**Reviewer #1:**

As a reviewer I have the following remarks.

1. Abstract: line 23. I suggest to use the term “sex” rather than “gender”.
**Response: Following the reviewer’s suggestion, we have replaced “gender” with “sex” in the main text.**

2. Line 25: “and age-groups (<18y, 18-59y, =60y).” We are guessing that “y” is for years? In Line 194 is OK.

**Response: “y” is for years. We apologize for this unclear statement in Line 25, which has been revised as “(<18, 18-59, ≥60 years)” (Line 26, Page 2).**

3. Line 29: “relative risk (RR) of 1.62 (95%CI: 1.45-1.80)” – in general, we don’t introduce any abbreviations if the terms are used only one. Thus we don’t need RR and 95%CI, rather use a full spell of them. “relative risk of 1.62 with the 95% confidence interval (1.45-1.80)” or something a similar.

**Response: We have revised this sentence** **as suggested (Lines 29-30, Page 2).**

4. Line 72. Is possible to provide ICD-10 codes for these diseases?

**Response: Thanks very much for the reviewer’s suggestion. We have added the ICD-10 code for DF “A90” in the revision (Lines 130-131, Page 7).**

5. Line 117. A trace of old reference style (24): “for 55.5% of the whole province’s population 24.”

**Response: We apologize for this mistake, which has been corrected in the revision (Line 120, Page 6).**

6. Line 174. Yes, reference [27] is correct for the case-crossover technique, published in 1991. It took time (2005) to develop and justify time-stratified approach: Janes H, Sheppard L, Lumley T. Case-crossover analyses of air pollution exposure data: referent selection strategies and their implications for bias. Epidemiology. 2005 Nov;16(6):717-26. doi: 10.1097/01.ede.0000181315.18836.9d.

**Response: Thanks very much for the reviewer’s comment. We have added this reference in the revision (Line 178, Page 9).**

7. Table 2. “Sex”.
**Response: Done (Line 220, Page 12).**

**Reviewer #2:**

This study examined the association between tropical cyclones and dengue fever in the Pearl River Delta of China, using data from 2013-1018.The main conclusions are that, Tropical cyclones may increase the risk of local transmission of dengue fever in south China, with the elderly more vulnerable. Generally, the paper is well written and all procedures and observations have been thoroughly explained in the discussion. There are some issues to be addressed:
**Response: We thank the reviewer for the positive feedback on our study and hope the following addresses the concerns sufficiently.**

1. Generalizability of this study and impact of the 2014 outbreak should be discussed.

**Response: According to the National Notifiable Disease Surveillance System (NDSS), about 70% of DF cases in China occurred in the PRD region during the study period. Our study therefore shows a reasonable representativeness at the national level. Following the reviewer’s great suggestion, we have discussed the generalizability as a strength of our study in the revision (Lines 343-345, Page 18):**

**‘According to the DF surveillance data of NDSS, about 70% of DF cases in China occurred in the PRD region during the study period. This high proportion of DF incidence promises a reasonable representativeness of our study at the national level.’**

**In this study, the majority of DF cases were from the 2014 outbreak. However, our use of time-stratified case-crossover design should help reduce the bias caused by a single outbreak, and describe the average association between tropical cyclones and DF incidence between 2013 and 2018.**

2. There are six levels of tropical cyclone. In this study, tropical storm and severe tropical storm were combined into tropical storm category, typhoon, severe typhoon and super typhoon were classified into typhoon category. What happened to tropical depression?

**Response: We apologize sincerely for this unclear statement. As shown in Table 1,** **during the study period there were no tropical cyclones with strength belonging to tropical depression at the landing time in the PRD. We have added an additional sentence to clarify our statement (Lines 153-155, Page 8):**

**‘During the study period there were no tropical cyclones with strength belonging to tropical depression at the landing time.’**

3. Line 63: “Within in China” should be “In China”.

**Response: Done (Line 62, Page 4).**

4. Line 64: “than any other locations” should be “than any other provinces”.

**Response: Done (Line 63, Page 4).**

5. Line 84-85: the first sentence in this paragraph seems not logical here.

**Response: Thanks for the reminding. We have removed this sentence from the revision (Line 84, Page 5).**

6. Line 172-173: “It eliminates the threat of control selection bias” better be replaced by “It reduces the selection bias”.

**Response: We have revised this sentence following the reviewer’s suggestion (Line 177, Page 9).**

7. Line 280: “elderly was more affected than other age subgroups.” better be replaced by “elderly was more vulnerable than other age subgroups.”.

**Response: We have revised this sentence following the reviewer’s suggestion (Line 276, Page 15).**

**Reviewer #3:**

Dengue fever is of great public health concern in China affecting a large number of people every year. Although there are ample evidence of weather changes and dengue fever incidence, knowledge is lacking as to the impacts of weather-related disasters on dengue fever incidence. To address this important scientific topic, this study makes full use of recent dengue fever data in China to examine the association between tropical cyclones and dengue fever incidence. This manuscript is well written with important and interesting findings being clearly reported. I’ve few minor comments for authors to consider.

**Response: We thank the reviewer for the positive feedback on our study and hope the following addresses the concerns sufficiently.**

#1: Study period could be shown in the title.

**Response: Following the reviewer’s great comment, we have added the study period information in the title (Line 2, Page 1).**

#2: Abstract section: The aim of this study is unclear. I guess the authors sought to focus on dengue fever “incidence”. If so, this point needs to be clear.

**Response: Thanks. Our study indeed focused on dengue fever incidence, and we have added the word “incidence” in the whole manuscript.**

#3: There are some grammar issues throughout this manuscript, which should be further polished up by a native English speaker.

**Response: Thanks. The revision has been polished by a native English speaker.**

#4: Lines 76-77: “As a typical imported disease…”, the meaning of this statement is unclear. Do you mean that dengue fever is a typical imported disease in China? Actually, most cases are local patients.

**Response: We apologize sincerely for this unclear statement. “imported disease” has been removed to reduce the confusion. This sentence has been revised as “As a tropical infectious disease…” (Lines 76-77, Page 4).**

#5: Potential mechanism of tropical cyclones and dengue fever incidence could be briefly introduced in the Introduction section.

**Response: Thanks. We have briefly described the possible mechanism of tropical cyclones and dengue fever in the Introduction section (Lines 88-93, Page 5):**

**‘****The transmission of DF is related to tropical cyclones via several potential pathways. The moderate to heavy rainfalls following tropical cyclones may increase mosquito breeding sites. Meanwhile, the destruction of tropical cyclones may increase the population’s displacement, their exposure risk to mosquito bites and delay in access to healthcare services.’**

#6: More information is needed to clarify the selection of the studied months from June to October. Does the risky season mean the period with the highest incidence of dengue fever?
**Response: June to October was selected as the study period because all tropical cyclones landed in the PRD during this period with DF incidence also substantial. We have revised this sentence to clarify our considerations (Lines 124-124, Page 7):**

**‘In this study, we chose June to October as the study period considering all tropical cyclones landed during this period and the meantime highest DF incidence in the PRD of the year.’**

**Reviewer #4:**

This study used time-stratified case-crossover study design to examine the association between tropical cyclones and dengue fever in the Pearl River Delta in China. In general, the article is logically and scientifically qualified with a certain innovation. Yet there are some issues need to be revised and addressed before accepted for publication.
**Response: We thank the reviewer for the positive feedback on our study and hope the following addresses the concerns sufficiently.**

1. In the Introduction (P5, Line 99), the authors mentioned using case-crossover design. As the methods part explained reasons of selecting the design, is it possible to add a few sentences describing using case-crossover design in previous studies (can be other vector borne diseases associated with cyclone and extreme weather) before express study aim? Thus the readers can better understand using the design even if there may be overlap with contents in Methods part.

**Response:** **We thank the reviewer very much for this valuable suggestion. The time-stratified case-crossover design has been widely used by epidemiological studies to explore the association between various meteorological risks and health outcomes (e.g., vector-borne diseases and other communicable diseases). We have added a brief introduction in the Introduction section so that the general readers may better understand this design (Lines 101-106, Page 6)****:**

**‘The case-crossover design has been widely used by epidemiological studies to explore the association between various meteorological risks and health outcomes (e.g., vector-borne diseases) [23-25]. It is especially suitable for exploring the effect of short-term exposure on acute events. The principle is that each patient is treated as a stratum, where the exposure during the risk period is compared with exposures during the control periods [26].’**

2. P7, 2.3 Disease data. The authors mentioned that the imported DF cases were excluded from this study. If the imported cases did not cause local transmission, this can be determined and sounds logically. The authors need to address it.

**Response: This study aimed to explore the association between tropical cyclones and local DF incidence. Although the imported DF cases indeed worked as the sources of dengue virus, these patients were infected outside the PRD region before tropical cyclones. We thus excluded these cases from the analyses.**

3. P7, 2.4 Tropical cyclones and meteorological data. As the intensity (Beaufort level) of each cyclone may be changed (usually weakened after landing) during the process (e.g., a few days). As this study used maximum value by Beaufort level to describe intensity and determined time lag, is there some literature to support it?

**Response: We apologize sincerely for our unclear statement. As with a previous study and the China Meteorological Administration, the Beaufort level was only used to define the exposure period of each tropical cyclone, i.e., the time interval from the date when the wind force circle at Beaufort level seven arrived the PRD region to the date when it left the PRD or disappeared. In this study, tropical cyclones were classified into six intensity levels according to the maximum wind velocity near the bottom of tropical cyclone center at the time of landing, which is also the official classification of the China Meteorological Administration. This classification strategy has been widely used by previous studies** **[1-3]. We have added the explanation aforementioned into the revision to make our statement clearer (Lines 149-158, Page 8).**

***References:***

**[1] Deng Z, Xun H, Zhou M, Jiang B, Wang S, Guo Q, et al. Impacts of tropical cyclones and accompanying precipitation on infectious diarrhea in cyclone landing areas of Zhejiang Province, China. Int J Environ Res Public Health. 2015;12(2):1054-68. https://doi.org/10.3390/ijerph120201054**

**[2] Kang R, Xun H, Zhang Y, Wang W, Wang X, Jiang B, et al. Impacts of different grades of tropical cyclones on infectious diarrhea in Guangdong, 2005-2011. PLoS One. 2015;10(6):e0131423. https://doi.org/10.1371/journal.pone.0131423**

**[3] Jiao K, Hu W, Ren C, Xu Z, Ma W. Impacts of tropical cyclones and accompanying precipitation and wind velocity on childhood hand, foot and mouth disease in Guangdong Province, China. Environ Res. 2019;173:262-9. https://doi.org/10.1016/j.envres.2019.03.041**

4. P15, Line 274-276 “This is one of the few studies exploring the relationship between tropical cyclones and DF worldwide. Meanwhile, this is the first study, to our knowledge, to explore the association in the PRD region, China.” This part can be relocated and integrated with strengths of this study.

**Response: We thank the reviewer very much for this valuable suggestion. We have integrated these sentences into the part of strengths (Lines 340-342, Page 18):**

5. P282-283: “For instance, a study in Southeast China documented that tropical cyclone was likely to increase the risk of DF”. In this study, besides located in PRD, what other difference/new findings can be addressed compared with the SE China study?

**Response:**

**1. In comparison to the SE China study, our study has some strengths in design:**

(1) **In the SE China study, the disaster periods included the exposure periods plus the maximum incubation period of DF. The control periods were defined as days with the same duration and the same days of week as those days of the exposure period, plus one or more weeks before the exposure period (unidirectional strategy). RR was calculated by simply comparing the numbers of DF cases between disaster period and reference period, without adjusting for a variety of confounders. In our study, the control periods were defined as the same days of the week in the same calendar month of the onset date (time-stratified strategy). This strategy helps minimize the influence of long-term trend, seasonality and “weekday effect” on the results.**
(2) **Moreover, our analysis additionally adjusted for the confounding effects of meteorological factors. As a result, our study could reasonably reflect the association between tropical cyclones and DF incidence.**

**2. Our study added to some new evidence/findings:**

(1) **Our study additionally revealed that there was substantial lag effect of tropical cyclones on DF incidence, which was overlooked by the SE China study.**
(2) **The stratified analyses indicated that the association by intensity grades of tropical cyclones and age-groups, with typhoon showing greater impact than tropical storm, and individuals over 60 years old more vulnerable than others.**

6. P285, “Réunion”, better to be revised as “Réunion in the south of Indian Ocean”.

**Response: Done (Lines 281-282, Page 15).**

7. In describing limitations, the authors mentioned that the study “did not consider the impact (should also have “of” here) heterogeneity of different landing sites and different tracks of each tropical cyclone on the DF in different cities”. If considering spatial difference of impact of tropical cyclone on the DF, wind direction should also be considered as it may cause different spatial pattern of climate and mosquito distribution, thus influence spatiotemporal pattern of DF. Even in study considering PRD as a single site, wind direction may also potentially influence the pattern of DF (e.g., incidence). And this should also be addressed in the limitation.

**Response: We appreciate the reviewer’s valuable comment. Unfortunately,** **we were unable to adjust for the potential effect of wind direction in the model due to the lack of data. We have acknowledged this as a limitation in the revision, which warrants further exploration when data will be available in the future (Lines 363-367, Page 19):**

**‘Furthermore, different wind direction of tropical cyclones may also cause various spatial patterns of local climate and mosquito distribution, thus potentially affecting the local transmission of DF. However, we were unable to adjust for the potential effect of wind direction in the model due to the lack of data. This issue warrants further exploration when data will be available in the future.’**

8. Some language errors/issues need to be corrected, e.g., P14, Line 258, “elderly” can be removed. The manuscript needs proofreading before being accepted.

**Response: Thanks. We have removed “elderly” (Line 256, Page 14). The revision has been proofread by a native English speaker.**

9. For figures, the resolution (dpi) should be increased.

**Response: Thanks very much for the reminding. We have increased the resolution of all figures to 600dpi. We further used the PACE tool provided by PLOS to ensure that our figures meet the technical requirements.**
